# Beta human papillomavirus 8 E6 allows colocalization of non-homologous end joining and homologous recombination repair factors

**DOI:** 10.1371/journal.ppat.1010275

**Published:** 2022-02-11

**Authors:** Changkun Hu, Taylor Bugbee, Dalton Dacus, Rachel Palinski, Nicholas Wallace

**Affiliations:** 1 Division of Biology, Kansas State University, Manhattan, Kansas, United States of America; 2 Kansas State Veterinary Diagnostic Laboratory, Kansas State University, Manhattan, Kansas, United States of America; 3 Department of Diagnostic Medicine/Pathobiology, Kansas State University, Manhattan, Kansas, United States of America; University of North Carolina at Chapel Hill, UNITED STATES

## Abstract

Beta human papillomavirus (β-HPV) are hypothesized to make DNA damage more mutagenic and potentially more carcinogenic. Double strand breaks (DSBs) are the most deleterious DNA lesion. They are typically repaired by homologous recombination (HR) or non-homologous end joining (NHEJ). HR occurs after DNA replication while NHEJ can occur at any point in the cell cycle. HR and NHEJ are not thought to occur in the same cell at the same time. HR is restricted to cells in phases of the cell cycle where homologous templates are available, while NHEJ occurs primarily during G1. β-HPV type 8 protein E6 (8E6) attenuates both repair pathways. We use a series of immunofluorescence microscopy and flow cytometry experiments to better define the impact of this attenuation. We found that 8E6 causes colocalization of HR factors (RPA70 and RAD51) with an NHEJ factor (activated DNA-PKcs or pDNA-PKcs) at persistent DSBs. 8E6 also causes RAD51 foci to form during G1. The initiation of NHEJ and HR at the same lesion could lead to antagonistic DNA end processing. Further, HR cannot be readily completed in an error-free manner during G1. Both aberrant repair events would cause deletions. To determine if these mutations were occurring, we used next generation sequencing of the 200kb surrounding a CAS9-induced DSB. 8E6 caused a 21-fold increase in deletions. Chemical and genetic inhibition of p300 as well as an 8E6 mutant that is incapable of destabilizing p300 demonstrates that 8E6 is acting via p300 destabilization. More specific chemical inhibitors of DNA repair provided mechanistic insight by mimicking 8E6-induced dysregulation of DNA repair in a virus-free system. Specifically, inhibition of NHEJ causes RAD51 foci to form in G1 and colocalization of RAD51 with pDNA-PKcs.

## Author summary

Our previous work shows that a master transcription regulator, p300, facilitates two major DNA double strand break (DSB) repair pathways: non-homologous end joining (NHEJ) and homologous recombination (HR). By degrading p300, beta genus human papillomavirus 8 protein E6 (8E6) hinders pDNA-PKcs resolution, an essential step during NHEJ. NHEJ and HR are known to compete, with only one pathway initiating repair of a DSB. NHEJ tends to be used in G1 and HR occurs in S/G2. Here, we show that 8E6 allows NHEJ and HR to initiate at the same break site. We show that 8E6 allows HR to initiate in G1, suggesting that NHEJ starts but fails before HR is initiated at the same DSB. Next generation sequencing of the region surrounding a CAS9-induced DSB supports our hypothesis that this dysregulation of DSB repair is mutagenic as 8E6 caused a 15- to 20-fold increase in mutations associated with a CAS9-induced DSB. These studies support the putative role of HPV8 infections in non-melanoma skin cancer development.

## Introduction

Beta genus of human papillomaviruses (β-HPVs) are frequently found in human skin [1,2]. HPV replication requires actively proliferating cells and the replication machinery of the host cells. This puts β-HPV infections in conflict with the cell cycle arrest associated with the repair of UV photolesions that frequently occur in skin [3–6]. Potentially as a mechanism to counter cell cycle arrest, some β-HPVs hinder the cellular response to DNA damage [7–9]. The E6 protein from β-HPV type 8 (8E6) dysregulates the cellular response to DNA damage by binding and destabilizing p300, a histone acetyltransferase that regulates transcription by chromosome remodelling [10–12]. p300 destabilization decreases expression of at least four DNA repair genes (ATM, ATR, BRCA1, and BRCA2) [7,13–15]. 8E6 also reduces ATM and ATR activation in response to UV [8]. This hinders UV damage repair, making UV-induced pyrimidine dimers more persistent and UV more likely to cause double stranded DNA breaks (DSBs) [14].

These DSBs are primarily repaired by two pathways, non-homologous end joining (NHEJ) and homologous recombination (HR). NHEJ can happen throughout the cell cycle but tends to occur during G1 and early S phase [16–19]. NHEJ initiation occurs when a DSB is sensed, and Ku70 and Ku80 bind DNA near the lesion [16,20]. DNA-dependent protein kinase catalytic subunit (DNA-PKcs) is then recruited to form a heterotrimer known as the DNA-PK holoenzyme. This allows autophosphorylation of DNA-PKcs at S2056 (pDNA-PKcs) and facilitates downstream steps in the pathway, including Artemis activation [21–23]. Artemis has both endonuclease and exonuclease activity that removes single stranded DNA, producing blunt ends [24–26]. Once processed, other NHEJ factors (e.g. XRCC4, XLF, and DNA ligase IV) ligate the gap to resolve the DSB [27–29].

HR uses a sister chromatid as a homologous template to provide error-free repair but is restricted to the S and G2 phases [19,30–32]. HR initiation includes the formation of a MRE11, RAD50, and NBS1 heterotrimer, known as the MRN complex [33]. The MRN complex together with CtIP, EXOI, and DNA-BLM complex resects DNA around the DSB, resulting in single stranded DNA overhangs [34–38]. An RPA trimer (RPA70, RPA32, and RPA14) coats and stabilizes this single stranded DNA [37,39]. Then BRCA1, BRCA2, and PALB2 facilitate the exchange of RPA trimers for RAD51 [19,40]. Finally, RAD51 facilitates a search for the homologous template, strand invasion, and resolution of the lesion [31,41,42].

8E6 attenuates both NHEJ and HR, by preventing the resolution of pDNA-PKcs (NHEJ) and RAD51 (HR) [7,9]. 8E6 also impairs DNA-PKcs activity. These repair defects are the result of 8E6 binding and destabilizing p300. These observations are consistent with the hypothesized ability of some β-HPV infections in promoting skin cancer, by making DNA damage more mutagenic [43,44]. Because β-HPV infections are generally transient, they are thought to induce mutations in premalignant lesion (actinic keratosis) that drive tumorigenesis independent of continued viral gene expression. This model is based in part on observations that β-HPV viral loads are higher in actinic keratosis than in cutaneous squamous cell carcinoma [45,46]. However, the feasibility of this “hit and run” model of tumorigenesis rest on the β-HPV expressing proteins (e.g., 8E6) that are sufficiently genotoxic to introduce tumorigenic mutations before the viral infection is cleared. Thus, to evaluate the potential pathogenicity of HPV8, our group has been characterizing the mutagenic potential of 8E6.

Here, we use immunoblotting, microscopy, and flow cytometry to characterize the persistent pDNA-PKcs and RAD51 repair complexes caused by 8E6 [7,9]. The DNA end processing during NHEJ and HR are mechanistically incompatible; therefore, NHEJ (pDNA-PKcs) and HR (RAD51) repair complexes are not often seen at the same break site [47]. We show that 8E6 promotes the colocalization of pDNA-PKcs and RAD51 foci. 8E6 also causes RAD51 foci to form during G1, when finding a homologous template will be unlikely. This dysregulated DSB repair is caused by the destabilization of p300. It can be phenocopied by chemical inhibition of the NHEJ pathway that mimics the NHEJ inhibition seen when p300 is destabilized. Finally, we developed an assay that combines the ability of a CAS9 endonuclease to be targeted to a specific genomic locus and next generation sequencing to define the extent to which 8E6 increased mutations during DSB repair. This approach demonstrated 8E6 caused a greater than 15-fold increase in overall mutations and a greater than 20-fold increase in deletions in the 200 kb surrounding a DSB.

## Results

### 8E6 promotes the recruitment of HR factors to sites of stalled NHEJ

To characterize the persistent NHEJ (pDNA-PKcs) and HR (RAD51) repair complexes associated with 8E6 expression, we used previously described vector control (HFK LXSN) and 8E6 (HFK 8E6) expressing telomerase (N/TERT) immortalized human foreskin keratinocyte cell lines [48]. 8E6 was HA-tagged and the expression was confirmed (S1A and S1B Fig). DSBs were induced by growth in media containing 10 μg/mL of zeocin for 10 min, a water-soluble radiation mimetic [49]. While UV is the most common mutagen encountered by cutaneous keratinocytes, zeocin was used to induce DSBs because 8E6 doubles the amount and delays the onset of UV-induced DSBs [14]. Thus, direct comparisons between UV-induced DSB repair would have required normalization to account for the differences in the time for DSBs to occur and the quantity of DSBs induced.

**Fig 1:**
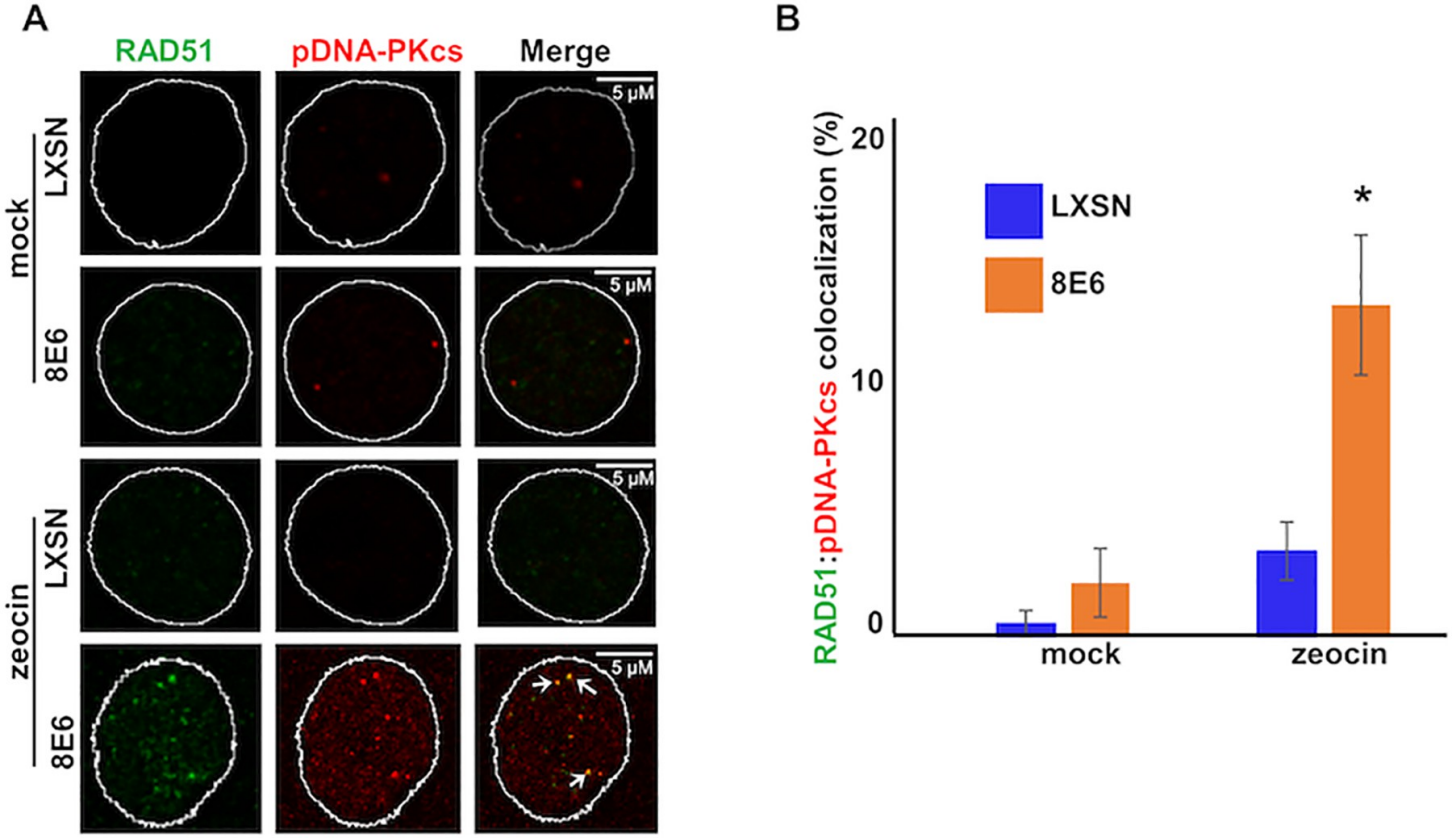
8E6 allows RAD51:pDNA-PKcs colocalization. (A) Representative images of HFK LXSN and HFK 8E6 cells stained for RAD51 (green) and pDNA-PKcs (red) 24 hours after DSB induction by growth in media containing zeocin (10 μg/mL, 10 min) or media containing additional water (solvent for zeocin) as a negative control. Additional water control is described as “mock” in Figure. White arrows indicate colocalizing RAD51 and pDNA-PKcs foci. (B) Percentage of HFK cells with colocalized RAD51 and pDNA-PKcs foci after mock treatment or zeocin treatment. All values are represented as mean ± standard error. The statistical significance of differences between cell lines were determined using Student’s t-test. * indicates significant difference between 8E6 and LXSN with same treatment (p< 0.05). At least 150 cells were counted over three independent experiments. Nuclei were determined by DAPI staining. The edge of this staining is shown by a white line depicting the nucleus.

Twenty-four hours after DSB-induction, RAD51 and pDNA-PKcs repair complexes were detected. Consistent with numerous reports that NHEJ and HR are employed at separate phases of the cell cycle [50–52], HFK LXSN cells less frequently contained both RAD51 and pDNA-PKcs foci (S1C and S1D Fig). However, HFK 8E6 cells were more likely to have both RAD51 and pDNA-PKcs foci in the same cells (S1C and S1D Fig). Moreover, 8E6 significantly increased the colocalization of RAD51 and pDNA-PKcs repair complexes (Fig 1A and 1B). These colocalizations occur at DNA break sites, as evidenced by co-staining with phosphorylated H2AX (serine 139, pH2AX) foci, a standard marker for DSBs (S1E Fig).

**Fig 2:**
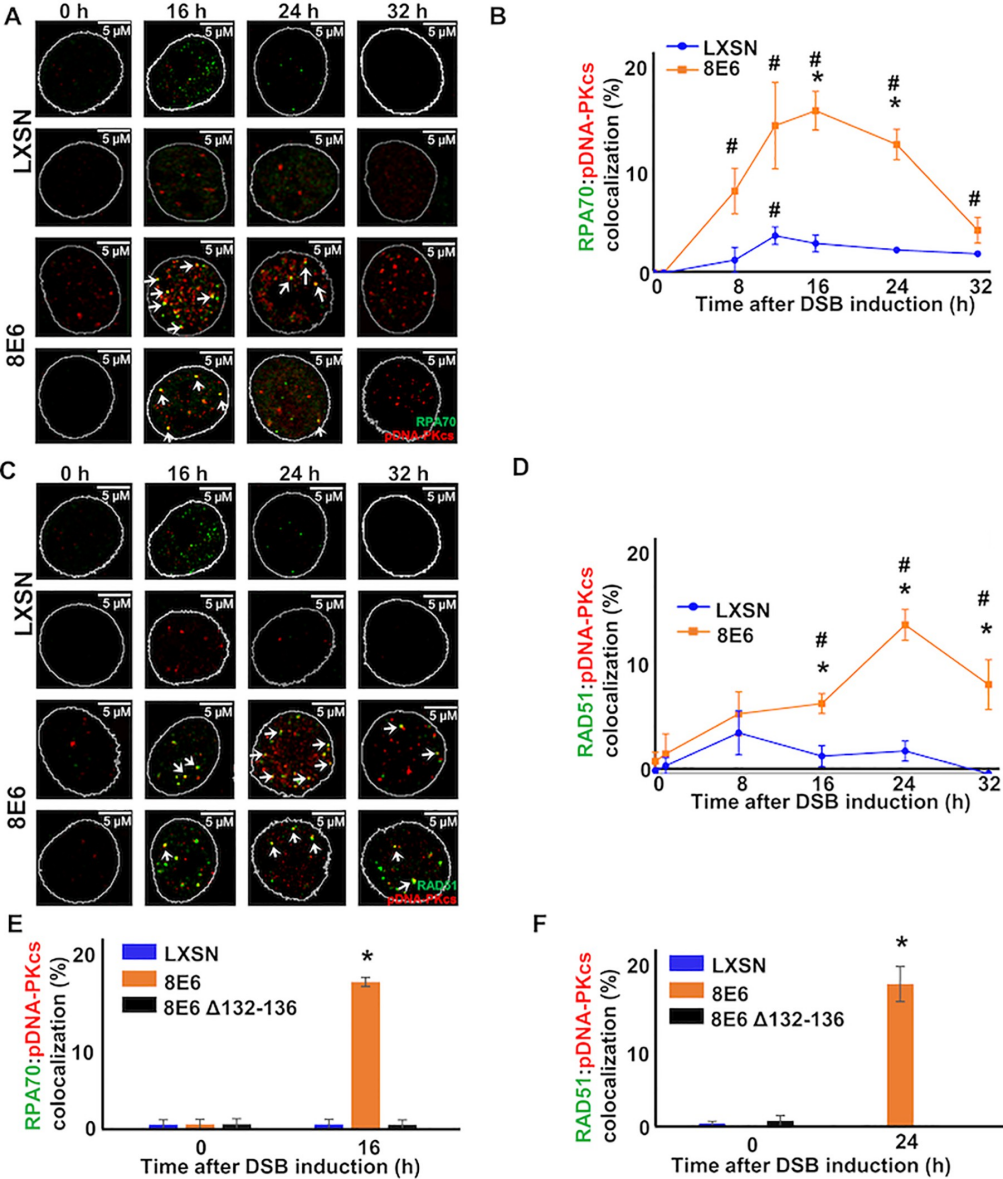
By binding p300, 8E6 allows RAD51 and RPA70 foci to colocalize with persistent pDNA-PKcs foci. (A) Representative images of HFK LXSN and HFK 8E6 cells stained for RPA70 (green) and pDNA-PKcs (red) following zeocin treatment (10 μg/mL, 10 min). (B) Percentage of HFK cells with colocalized RPA70 and pDNA-PKcs foci. (C) Representative images of HFK LXSN and HFK 8E6 cells stained for RAD51 (green) and pDNA-PKcs (red) following zeocin treatment (D) Percentage of HFK cells with colocalized RAD51 and pDNA-PKcs foci. (E) Percentage of primary HFKs with colocalized RPA70 and pDNA-PKcs foci. (F) Percentage of primary HFKs with colocalized RAD51 and pDNA-PKcs foci. White arrow indicates colocalization. All values are represented as mean ± standard error. The statistical significance of differences between two groups were determined using Student’s t-test. * indicates significant difference between 8E6 and LXSN with same treatment (p< 0.05). # indicates a significant difference (p < 0.05) between control (solvent) and treated group within each cell line. At least 150 cells were counted over three independent experiments. Nuclei were determined by DAPI staining. The edge of this staining is shown by a white line depicting the nucleus.

To better characterize this colocalization, we observed the colocalization of RAD51 and pDNA-PKcs foci in HFK LXSN and HFK 8E6 over a 32-hour period after DSB-induction. We also determined the extent to which another HR factor (RPA70) colocalized with pDNA-PKcs. RPA70 repair complex formation occurs immediately prior to RAD51 complex formation during HR. In LXSN cells, the colocalization of RPA70 and RAD51 with pDNAPKcs was rarely observed with or without damage (Fig 2A–2D). In contrast, both RPA70:pDNA-PKcs and RAD51:pDNA-PKcs colocalization were significantly increased in HFK 8E6 cells compared to untreated controls and similarly treated HFK LXSN cells (Fig 2A–2D). If repair complexes are not resolved, they increase in size with time, making them appear more intense when detected by microscopy [53]. Consistent with the idea that colocalized repair complexes represent difficult to repair DSBs, RAD51 and pDNA-PKcs foci were more intense when colocalized (S2 Fig). We also compared the kinetics of RPA70 and RAD51 colocalization. During canonical HR, RPA complexes are replaced by RAD51. Consistent with active progression through the canonical HR pathway, the peak for RPA70:pDNA-PKcs foci is followed by the peak for RAD51:pDNA-PKcs colocalization (Fig 2A–2D). Supporting the biological relevance of our work, 8E6 increased RAD51:pDNA-PKcs colocalization in response to UV damage (S3 Fig).

Because we have previously reported that exogenous TERT expression can augment 8E6-associated genome destabilization [54], we measured colocalization in primary keratinocytes expressing 8E6 or vector control (primary HFK 8E6 and primary HFK LXSN, respectively). p300 degradation by 8E6 (relative to LXSN) was confirmed by immunoblot (S4A Fig). 8E6 continued to cause a significant increase in both RPA70 and RAD51 colocalization with pDNA-PKcs in these cells (Fig 2E and 2F). Because 8E6 has been reported to dysregulate DSB repair by destabilizing p300, we also expressed a previously described mutant of 8E6 that cannot destabilize p300 (8E6 Δ132–136) in primary HFKs (primary HFK 8E6 Δ132–136). The inability of 8E6 Δ132–136 to reduce p300 levels (relative to LXSN) was confirmed by immunoblot (S4A Fig). We did not detect elevated levels of RAD51 or RPA70 colocalization with pDNA-PKcs in primary HFK 8E6 Δ132–136 (Fig 2E and 2F). S5 Fig describes the frequency of primary HFK LXSN, 8E6, and 8E6 Δ132–136 cells with no foci; just RPA70, RAD51, or pDNA-PKcs foci; with HR and NHEJ foci in the same cell but not colocalized, and cells with colocalization of HR and NHEJ factors. This analysis also demonstrated that the majority of RAD51 foci present in HFK 8E6 cells 24 hours after zeocin were colocalized with pDNA-PKcs. Together, our data shows that 8E6 causes colocalization independently of exogenous TERT expression. They also suggest that 8E6 acts by destabilizing p300.

### p300 catalytic activity prevents colocalization of HR factors with persistent pDNA-PKcs repair complexes

**Fig 3:**
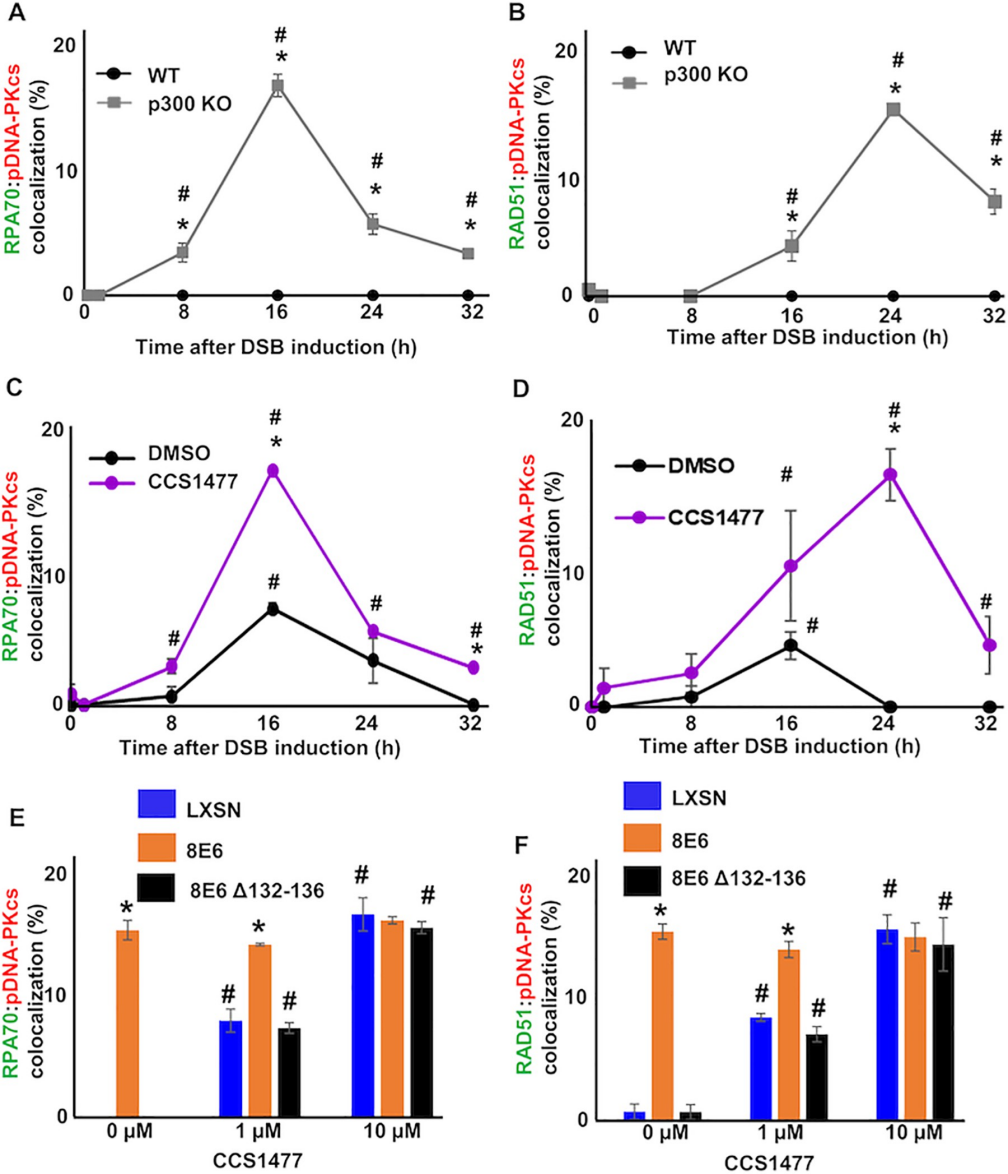
p300 reduces co-localization of RAD51 and RPA70 foci with pDNA-PKcs foci. (A-B) Percentage of HFK WT and p300 KO cells with (A) colocalized RPA70 (green) and pDNA-PKcs (red) foci or (B) RAD51 (green) and pDNA-PKcs (red) foci over a 32-hour time course following zeocin exposure (10 μg/mL, 10 min). (C-D) Percentage of CCS1477 (1 μM) or DMSO treated HFK LXSN cells that contained colocalized (C) RPA70 (green) and pDNA-PKcs (red) or (D) RAD51 (green) and pDNA-PKcs (red) foci following zeocin treatment. (E-F) Percentage of primary HFK cells treated with CCS1477 or DMSO that contained (E) colocalized RPA70 and pDNA-PKcs foci 16-hours following zeocin treatment or (F) RAD51 and pDNA-PKcs foci 24-hours following zeocin treatment. All values are represented as mean ± standard error. The statistical significance of differences between cell lines or treatments were determined using Student’s t-test. * indicates significant difference between two groups (A-D) or LXSN and 8E6 (E-F) with same treatment (p< 0.05). # indicates p < 0.05 between control (0 h) and zeocin treated group within each cell line. At least 150 cells were counted over three independent experiments.

Because deletion of the p300 binding domain has been reported to disrupt other functions of 8E6 [55], data obtained from 8E6 Δ132–136 must be further verified. To this end, we used CRISPR/CAS9 technology to knock out p300 in telomerase-immortalized human foreskin keratinocyte cell lines (N/TERT HFKs) that we will refer to as HFK p300 KO. A non-targeting control was used to produce a control cell line (HFK p300 WT). The knockout of p300 was confirmed by immunoblot (S4B Fig). We detected RPA70:pDNA-PKcs and RAD51:pDNA-PKcs colocalization in these cells over a 32-hour period after DSB induction. Demonstrating a role for p300 in preventing colocalization of HR and NHEJ factors, there was a significant increase of both RPA70 and RAD51 colocalizing with pDNA-PKcs in HFK p300 KO compared to HFK p300 WT cells (Fig 3A and 3B).

p300 is a large protein (~300kDa) that can promote repair by acting as a scaffold for other repair factors and through its catalytic activity [56]. To determine whether p300 prevented HR:NHEJ colocalization by acting as a scaffold or through its catalytic activity, HFK LXSN cells were grown in media containing a small molecule (1 μM of CCS1477) that blocks p300 catalytic activity. Because our previous work demonstrated that p300 is required for ATM and ATR activation [8], damage-induced ATM and ATR phosphorylation were used as a positive control for p300 inhibition. Immunoblots demonstrated that CCS1477 reduced ATM and ATR phosphorylation (S6 Fig). CCS1477 significantly increased RPA70:pDNA-PKcs and RAD51:pDNA-PKcs colocalization (Fig 3C and 3D). Again, the colocalization of RPA70:pDNA-PKcs peaked before colocalization of RAD51:pDNA-PKcs. CCS1477 also increased this colocalization in primary HFK LXSN cells, demonstrating that the effect is independent of exogenous TERT expression (Fig 3E and 3F). As a further control that 8E6 was acting by reducing p300 activity, we measured colocalization in primary HFK 8E6 and 8E6 Δ132–136 cells with CCS1477 (Fig 3E and 3F). Validating the p300-dependent mechanism of action, CCS1477 increased colocalization in primary HFK 8E6 Δ132–136 but not primary HFK 8E6 cells. This also supports the specificity of CCS1477.

### 8E6 allows RAD51 foci formation in G1 by binding to p300

**Fig 4:**
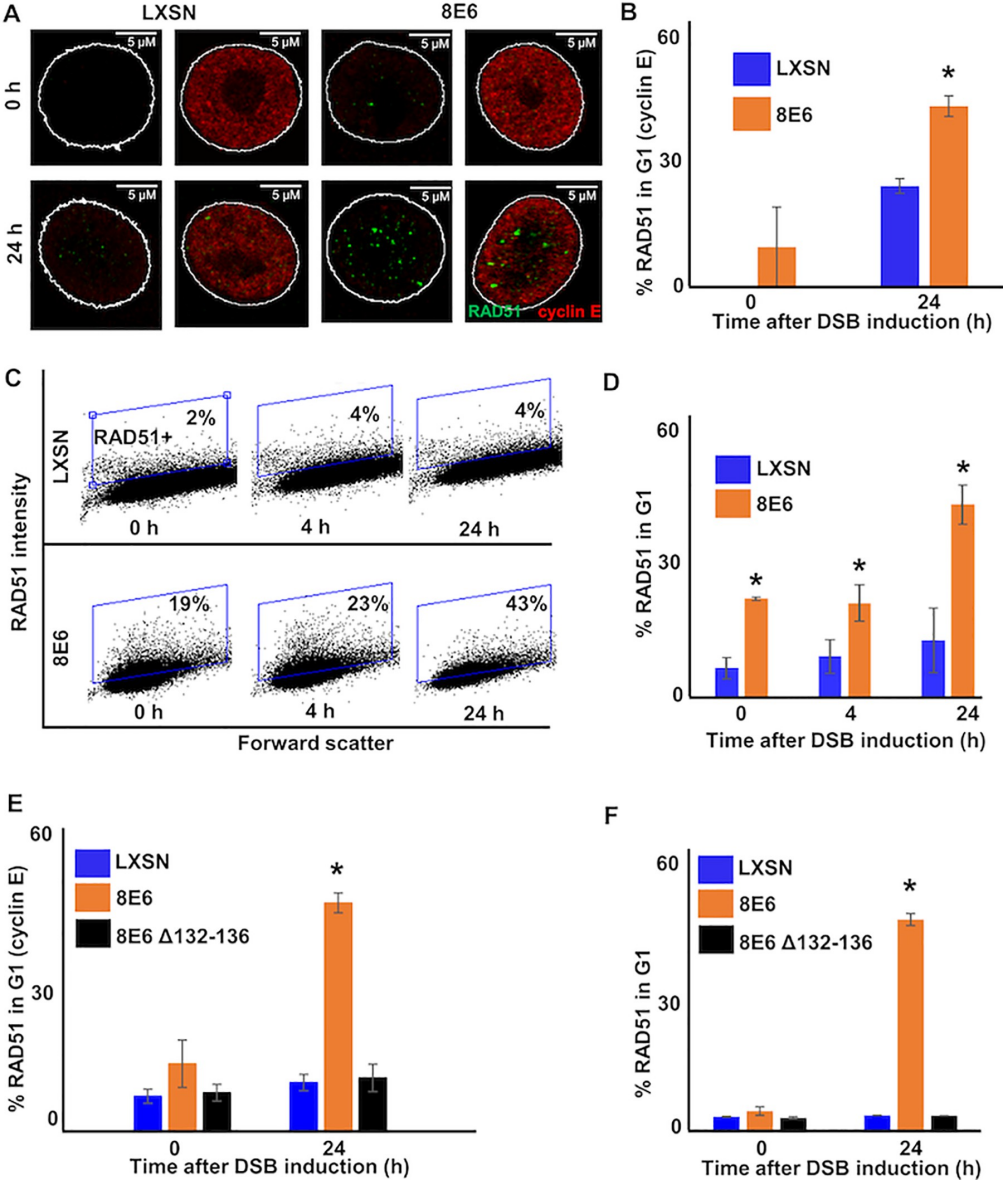
8E6 allows RAD51 foci formation in G1. (A) Representative cyclin E negative and positive HFK LXSN and HFK 8E6 cells stained for RAD51 (green) and cyclin E (red) 0 and 24 hours following zeocin treatment (10 μg/mL, 10 min). (B) Percentage of RAD51 positive HFK cell in G1 determined by cyclin E staining after zeocin treatment. (C) Representative images of flow cytometry results of HFK LXSN and HFK 8E6 cells in G1 stained with RAD51 at three points after zeocin exposure (0, 4, and 24 hours). RAD51 intensity is determined by Alexa 488 conjugated secondary antibody and shown on the y-axis. The gating represents RAD51 positive based off secondary only control. The x-axis shows cells distributed by forward scatter to avoid debris. (D) Percentage of HFK cells in G1 that are positive for RAD51 as determined by flow cytometry. (E-F) Percentage of primary HFKs in G1 that RAD51 staining after zeocin treatment as determined by (E) Cyclin E staining or (F) flow cytometry. All values are represented as mean ± standard error. The statistical significance of differences between LXSN and 8E6 cell lines were determined using Student’s t-test. * indicates significant difference between 8E6 and LXSN with same treatment (p< 0.05). At least 150 cells were counted over three independent experiments for microscopy. Twenty thousand cells were counted for each of three independent flow cytometry experiments. Nuclei were determined by DAPI staining. The edge of this staining is shown by a white line depicting the nucleus.

The colocalization of pDNA-PKcs with HR factors (RPA70 and RAD51) associated with sequential steps in HR indicates that progression through the HR pathway occurs when NHEJ stalls. If this is the case, then 8E6 likely allows HR factors to form repair complexes during G1. To test this, we used cyclin E as a marker of G1 [57,58]. Twenty-four hours after DSB induction, co-staining of cyclin E and RAD51 foci were significantly more frequent in HFK 8E6 than in HFK LXSN cells (Fig 4A and 4B). As cyclin E also occurs in early S, we used cyclin A as a marker of S/G2. HFK 8E6 increased the frequency of cyclin A negative cells with RAD51 foci compared to HFK LXSN (S7 Fig). Similar results were obtained by using flow cytometry of DAPI stained cells to identify cells in G1 (Figs 4C and 4D and S8A). The cut-off for RAD51 positivity was determined by measuring staining intensity after incubation with only the relevant secondary antibody (S8B Fig). Consistent with prior reports [59,60], RAD51 staining was infrequently detected in HFK LXSN cells during the G1. The increased RAD51 in G1 cells seen in HFK 8E6 cells was dependent on CtIP (S9 Fig). This was expected based on prior reports that CtIP was required for RAD51 repair complex formation in G1 in 53BP1 depleted cells [61]. Of note, 8E6 also promoted RAD51 foci formation during G1 in primary HFK cells (Fig 4C and 4D).

**Fig 5:**
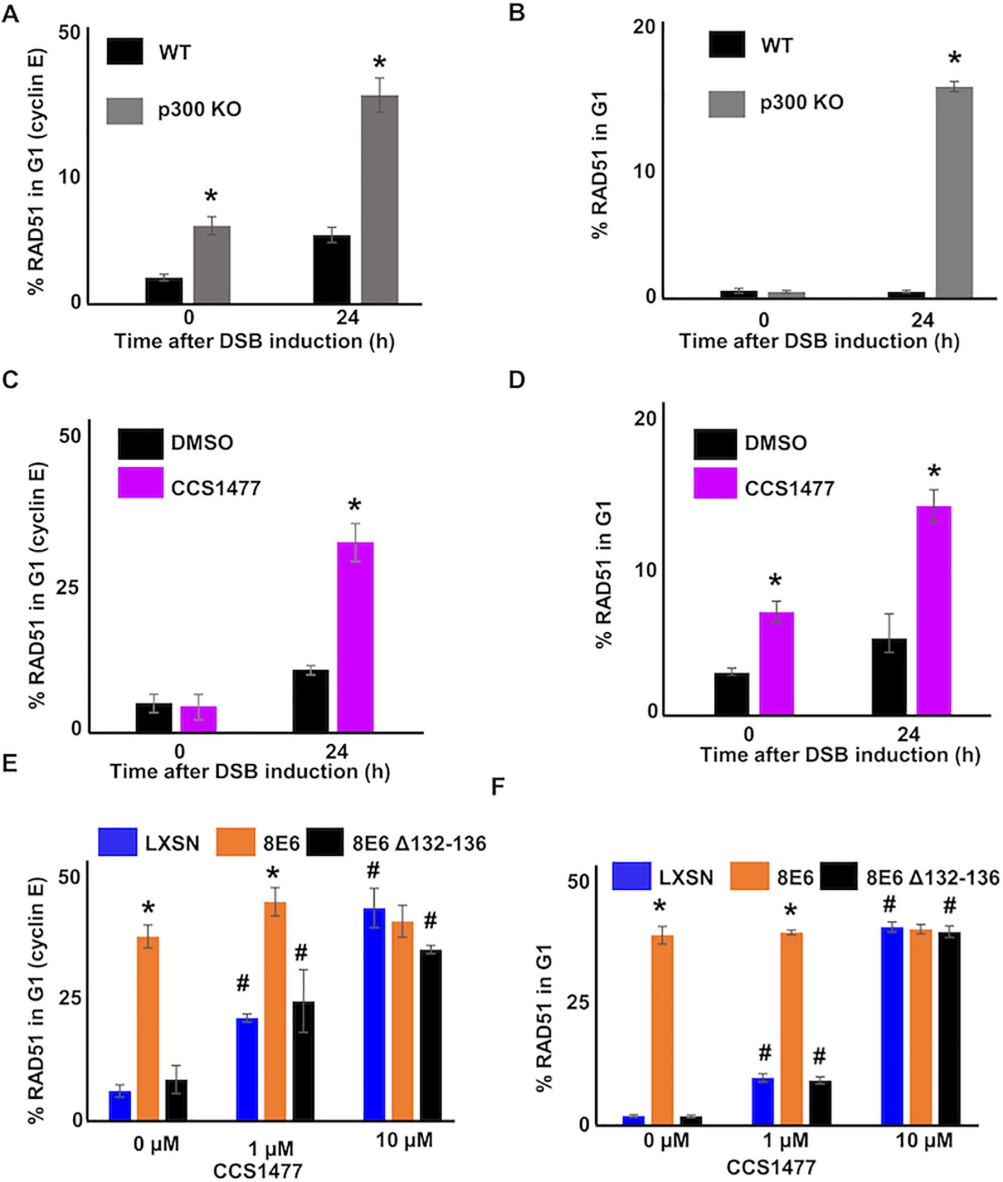
p300 restricts RAD51 foci formation in G1. (A-B) Percentage of HFK WT and HFK p300 KO cells with RAD51 staining in G1 as determined by (A) Cyclin E staining or (B) flow cytometry after zeocin treatment (10 μg/mL, 10 min). (C-D) Percentage of HFK LXSN cells treated with DMSO or CCS1477 (1 μM) that had RAD51 staining in G1 as determined by (C) Cyclin E staining or (D) flow cytometry after zeocin treatment. (E-F) Percentage of primary HFK LXSN cells treated with DMSO or CCS1477 that had RAD51 staining in G1 as determined by (E) Cyclin E staining or (F) flow cytometry after zeocin treatment. All values are represented as mean ± standard error. The statistical significance of differences between cell lines or treatments were determined using Student’s t-test. * indicates significant difference between two groups (A-D) or LXSN and 8E6 (E-F) with same treatment (p< 0.05). # indicates p < 0.05 between control (solvent) and treated group within each cell line. At least 150 cells were counted over three independent experiments for microscopy. Twenty thousand cells were counted for each of three independent flow cytometry experiments.

Consistent with a p300-dependent mechanism of action, HFK 8E6 Δ132–136 did not increase RAD51 complex staining in G1 cells (Fig 4E and 4F). Moreover, HFK p300 KO cells had increased RAD51 complex staining in G1 (Fig 5A and 5B). CCS1477 significantly increased the frequency of RAD51 staining in G1 in HFK LXSN, primary HFK LXSN, and primary HFK 8E6 Δ132–136 cells (Fig 5C–5F). However, CCS1477 was not able to further increase RAD51 staining in G1 in primary HFK 8E6 cells (Fig 5E and 5F). Together these data demonstrate that 8E6 promotes RAD51 repair complex formation during G1 by destabilizing p300.

### NHEJ inhibition leads to HR initiation in G1 and colocalization of RAD51 and pDNA-PKcs

**Fig 6:**
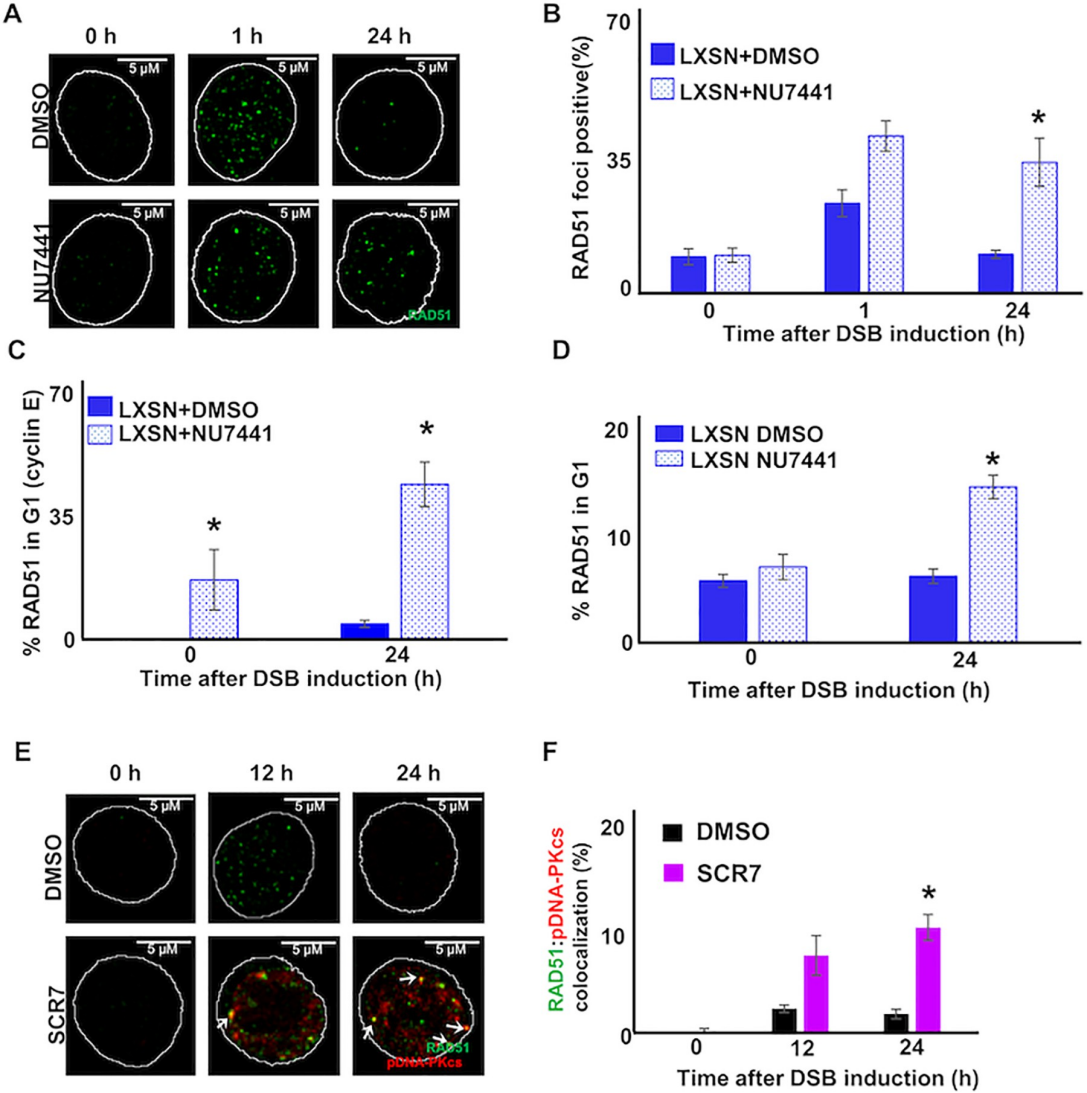
DNA-PKcs inhibition increases RAD51 foci in G1. (A) Representative images of HFK LXSN cells treated with NU7441 (1 μM) or DMSO stained for RAD51 (green) following zeocin treatment (10 μg/mL, 10 min). (B) Percentage of HFK LXSN cells treated with NU7441 (1 μM) or DMSO stained with RAD51 foci following zeocin treatment. (C-D) Percentage of HFK LXSN cells treated with NU7441 (1 μM) or DMSO stained with RAD51 staining in G1 as determined by (C) cyclin E staining or (D) flow cytometry. (E) Representative images of HFK LXSN cells treated with ligase IV inhibitor (1 μM of SCR7) or DMSO stained for RAD51 (green) and pDNA-PKcs (red) following zeocin treatment. (F). Percentage of HFK LXSN cells with colocalized RAD51 and pDNA-PKcs foci after treatment with ligase IV inhibitor or DMSO and exposure to zeocin. White arrow indicates colocalization. All values are represented as mean ± standard error. The statistical significance of differences between treatments were determined using Student’s t-test. * indicates significant difference between control (DMSO) and inhibitor treated groups with same zeocin treatment (p < 0.05). At least 150 cells were counted over three independent experiments for microscopy. Twenty thousand cells were counted for each of three independent flow cytometry experiments. Nuclei were determined by DAPI staining. The edge of this staining is shown by a white line depicting the nucleus.

8E6 impairs multiple DSB repair mechanisms. Our prior work demonstrated that 8E6 hindered DNA-PKcs activity. Our data also suggest that 8E6 caused HR factors to be recruited to sites of stalled NHEJ repair. As a result, we treated HFK LXSN cells with a DNA-PKcs inhibitor (1 μM of NU7441) to mimic 8E6-mediated inhibition of NHEJ. We performed two controls to ensure that the DNA-PKcs inhibitor was working. As expected, the inhibitor blunted damage-induced autophosphorylation of DNA-PKcs and delayed the resolution of pH2AX foci (S10 Fig). DNA-PKcs inhibition also delayed the resolution of RAD51 foci and increased the frequency of RAD51 staining in G1 cells (Fig 6A–6D). We turned to a DNA ligase IV inhibitor (1μM of SCR7) that impaired the pathway after pDNA-PKcs foci formed [18]. Treating cells with SCR7 resulted in a significant increase in RAD51:pDNA-PKcs colocalization (Fig 6E and 6F). The most parsimonious explanation of these data is that 8E6 increases the formation of aberrant RAD51 foci (colocalized with pDNA-PKcs and present in G1) by causing NHEJ to stall. However, it is possible that 8E6 promotes colocalization in another manner.

### 8E6 increases genomic instability during DSB repair

**Fig 7:**
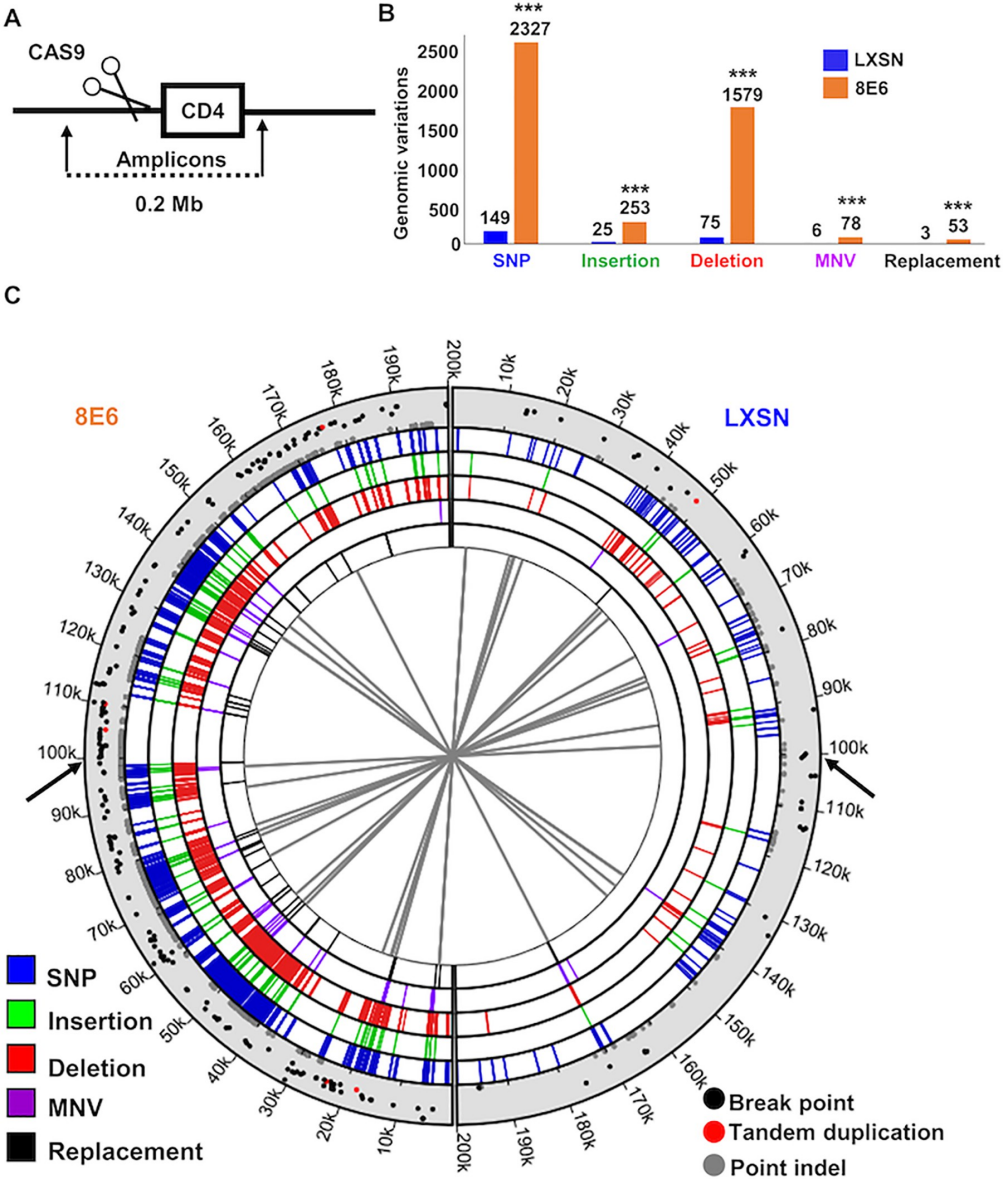
Beta-HPV 8E6 increases genomic instability during DSB repair. (A) Schematic of the placement of CAS9 induced DSB along the sequenced portion of the genome. (B) Genomic variations grouped by types of mutational events in HFK LXSN and HFK 8E6. Each group of genomic variations and total number of variations were compared between HFK LXSN and HFK 8E6. (C) Circos plot of DNA mutations in HFK LXSN (right side) and HFK 8E6 cells (left side). Black arrows indicate CAS9 cutting sites. The innermost circle displays connections between identical genomic rearrangements. The location of genomic rearrangements colored by types of genomic variations are shown in five concentric circles (blue represents SNP, green represents insertion, red represents deletion, purple represents MNV, and black represents replacement). Scatter plot in the outermost circle displays breakpoints (black), tandem duplications (red), and point indels (grey), in which proximity to the outer edge represents high variant ratio. SNP, single nucleotide polymorphism. MNV, multi-nucleotide variation. Statistical differences between cell lines were measured using a Students’ T-test. *** indicates p < 0.001.

To determine the extent to which 8E6 makes DSB repair more mutagenic, we transfected HFK LXSN and HFK 8E6 cells with vectors that expressed CAS9 endonuclease and sgRNA designed to induce a DSB just upstream of the CD4 open reading frame. Our group and others have previously described and validated this method of DSB induction [9,62]. We confirmed that this transfection induced RAD51:pDNA-PKcs colocalization in HFK 8E6, but not in HFK LXSN cells (S11A Fig). We designed a series of overlapping primers targeting the 100 kb region upstream and downstream of the CAS9 target site (Fig 7A). We then pooled the primers and used them to produce amplicons for next-generation sequencing. The resulting raw reads were trimmed for quality, mapped to the reference sequence, and assessed for mutations (SNPs, indels). As an additional control, 500 kb was sequenced surrounding the CAS9 cleavage site. Mutations within 100 kb of the CAS9 cleavage site were ~10-fold more common compared to sequences more distant from the CAS9 cleavage site (S11B Fig). These data showed a ~10-fold increase in mutations associated with a DSB in HFK 8E6 cells compared to HFK LXSN cells (Fig 7B–7C). This includes more replacements, insertions, deletions, multi-nucleotide variations, and single nucleotide polymorphisms (SNPs). Of these, deletions occur about 21-fold more often in HFK 8E6 than in HFK LXSN cells.

## Discussion

We have previously shown that 8E6 decreases the efficiency of HR and NHEJ [7,9]. This results in persistent pDNA-PKcs and RAD51 repair complexes and is dependent on 8E6 binding and destabilizing p300. Here, we demonstrate that 8E6 triggers HR repair factors (RPA70 and RAD51) to be recruited to DSBs when pDNA-PKcs repair complexes do not efficiently repair the lesion. Further, 8E6 allows RAD51 repair complexes to form in G1. These abnormal repair events are the result of p300-destabilization by 8E6 and the resulting inhibition of NHEJ after pDNA-PKcs activation. Finally, we developed an assay that uses targeted next generation sequencing at a CAS9-induced DSB to demonstrate that 8E6 significantly increases the frequency of mutations associated with DSB repair. Here, we discuss our interpretation of these results.

**Fig 8:**
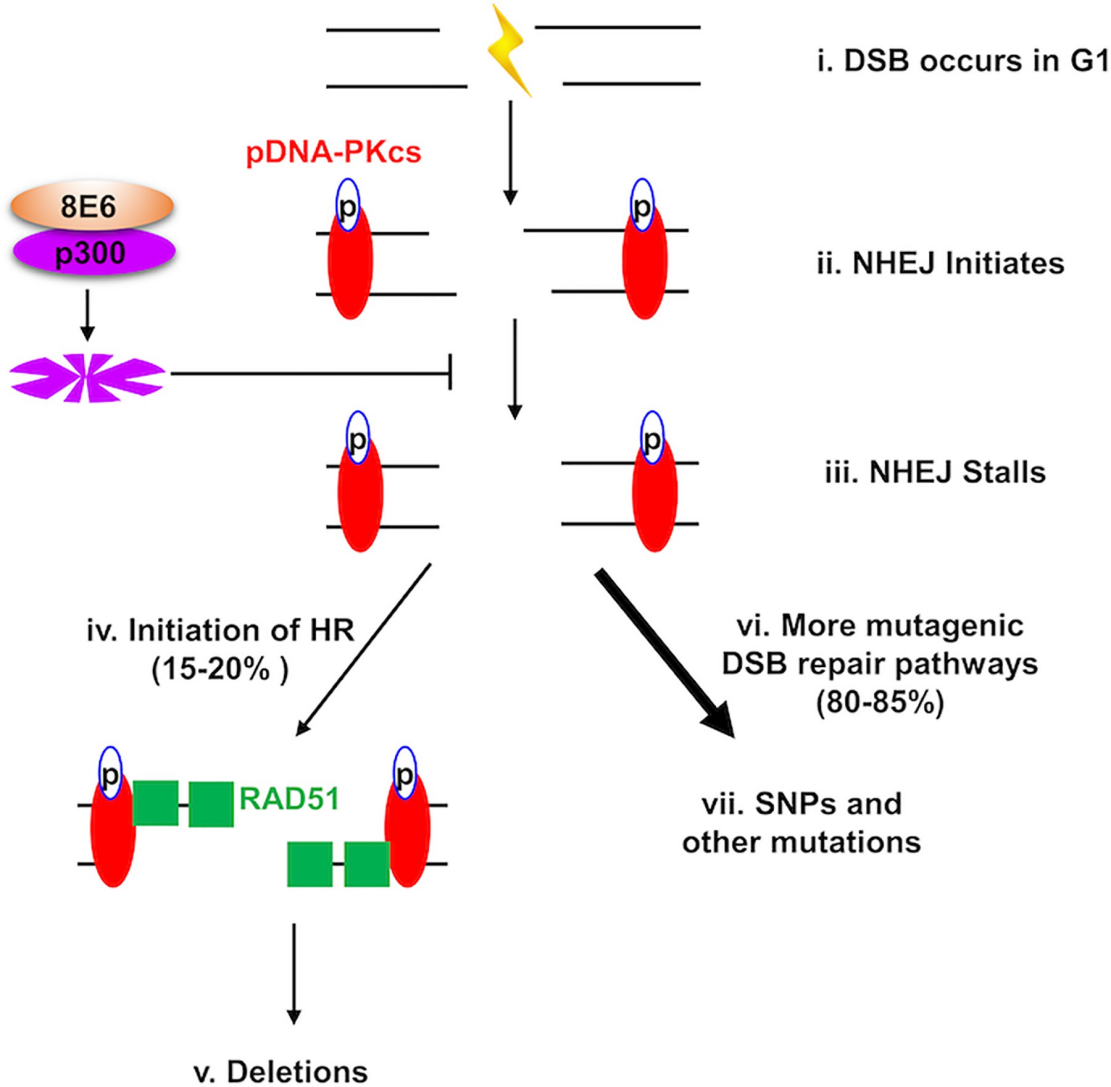
Summary figure of DSB repair in G1 phase. (i) DSB occurs in a cell in G1 phase that is expressing 8E6. (ii) NHEJ initiates. DNA-PKcs is recruited to the lesion where it is auto-phosphorylated. (iii) NHEJ stalls due to 8E6 mediated p300 degradation. This leaves unresolved pDNA-PKcs repair complexes [9]. (iv) HR initiates at the site of failed NHEJ. The MRN complex, CtIP, and EXO1 resect one strand of DNA near the lesion, producing single stranded DNA [73]. RPA complexes coat and stabilize the resulting single stranded DNA [39]. RPA70 foci colocalize with pDNA-PKcs indicating that HR-mediated DNA resection occurs after NHEJ fails. Then, RAD51 is recruited to the break site. (v) HR cannot be complete due to the lack of a homologous template and/or 8E6-mediated inhibition of HR [7]. This causes deletions due to antagonist DNA end process by NHEJ and HR and/or by failure to complete HR after resection. (vi) The failure of NHEJ causes cells to use tertiary DSB repair pathways to fix the lesion. (vii) This alternative repair pathway (e.g., Alt-EJ) is error prone and increases other types of mutations (such as SNPs).

We propose three ways in which 8E6-induced abrogation of DSB repair results in the increased mutations seen by our next generation sequencing analysis. They are depicted in Fig 8, described below, and notably are not mutually exclusive. Our data show that 8E6 does not prevent initiation of NHEJ as indicated by the formation of pDNA-PKcs repair complex formation. We have previously shown that 8E6 causes NHEJ to stall after pDNA-PKcs foci formation by destabilizing p300, hindering further DNA-PKcs activity and progression through the pathway [9]. The data presented here show that this results in progression through the HR pathway at sites of stalled NHEJ. Specifically, DNA near the DSB is resected (indicated by RPA70 foci formation) and RAD51 repair complexes form. This is expected to cause deletions in at least two ways. Since NHEJ occurs primarily during G1, the RAD51 foci that form in response to stalled NHEJ would be unlikely to find suitable homologous template to facilitate their resolution. As a result, the single stranded DNA (ssDNA) created by resection will be subject to degradation. Further, the initiation of NHEJ and HR at the same lesion is expected to result in antagonistic end processing that will result in deletions (NHEJ removes ssDNA, while HR generates ssDNA). Finally, the inability of cells to efficiently repair DSBs using HR and NHEJ likely shunts repair in more mutagenic tertiary repair pathways.

8E6 induced RAD51:pDNA-PKcs colocalization in response to low dose UV exposure. These data support our hypothesis that 8E6 leads to abnormal repair events in response to the levels of UV regularly encountered by cells infected with HPV8. We acknowledge that the reported RAD51:pDNA-PKcs could result from multiple DSBs occurring so close to each other that our microscopy cannot distinguish them as separate lesions. While we cannot formally exclude this possibility, it is unlikely. If the colocalization was the result of staining occurring at two separate DSBs, the maxima for colocalization would be expected when RAD51 and pDNA-PKcs staining is the highest. However, while RAD51 and pDNA-PKcs staining peak within a few hours of DSB induction, RAD51:pDNA-PKcs colocalization peaks 24 hours after DSB induction. Thus, we believe it is unlikely that the RAD51:pDNA-PKcs colocalization that we report is the result of staining of clustered DSBs.

There have been other reports of RAD51:pDNA-PKcs colocalization and resection in G1. One study found that RAD51:pDNA-PKcs colocalization occurs following a high dosage of UV radiation [63]. Another study found RAD51:pDNA-PKcs colocalization at common fragile sites in cells exposed to aphidicolin, a DNA polymerase inhibitor [64]. Given the complex nature of these lesions, the results are consistent with our hypothesis that co-localization of these factors occurs primarily at difficult to repair DSBs. However, unlike these studies we provide mechanistic insight into how RAD51:pDNA-PKcs colocalization occurs. Resection (indicated by RPA foci) has also been reported during G1 [38]. Biehs et al found that RPA complex formation during G1 can lead to Alt-EJ. Our data do not rule out that 8E6 may promote Alt-EJ. In fact, it seems likely that Alt-EJ plays a role in repairing DSBs in 8E6 expressing cells. However, some of our data were incongruent with other data reported by Biehs et al. Their study found that BRCA1 was required for robust RPA foci formation, while we have previously reported that RPA foci levels are not altered by 8E6-mediated reduction of BRCA1 [7]. We believe that differences in cell culture models likely explain the inconsistencies. We used primary and TERT-immortalized keratinocytes, while Biehs et al used mouse embryonic fibroblasts (MEFs) and HeLa cells. HPV does not infect fibroblasts. Further, HeLa cells are immortalized by high-risk alpha-papillomavirus (HR-α HPV) oncogenes. These oncogenes have a well-documented ability to dysregulate both cell cycle and DSB repair [65]. Thus, the requirement of BRCA1 for RPA repair complex formation in cell systems relevant to β-HPV biology have has not been rigorously established elsewhere. Another relevant paper reported that extensive genetic manipulation (depletion of 53bp1 and KEAP1 along with the introduction of phospho-mimetic mutations in CtIP) was required for RAD51 foci to form during G1 [61]. This is consistent with our reports that 8E6 leads to extensive alteration of DSB repair [7,9].

The ability of high-risk alpha-papillomavirus (HR-α HPV) oncogenes to cause co-localization of HR and NHEJ factors has not been reported. However, the ability of these oncogenes to dysregulate DSB repair is an area of active investigation. Both HR-α HPV E6 and E7 have been shown to increase expression and post-translational modification of DSB repair proteins [66,67]. The additional repair factors facilitate the viral lifecycle as they are recruited away from damaged host DNA and to sites of viral replication [58,68]. HR-α HPV E7 has also been reported to decrease NHEJ [69]. Our data suggests that this reduction could result in the recruitment of HR factors to sites of failed NHEJ. Supporting this possibility, RAD51 foci have also been reported in G1 in Hela cells (transformed by HR-α HPV oncogenes) [70].

Our data are consistent with the proposed role of β-HPV infections in early stages of non-melanoma skin cancer development via genome destabilization. We show that 8E6 significantly increases the mutational burden of DSBs. However, we have only examined the E6 from HPV8. The E6 from some other members of the β-HPV genus do not destabilize p300, but can immortalize primary cells in combination with expression of β-HPV E7 [55]. Thus, continued investigations into the diversity of β-HPV biology are needed to fully evaluate the oncogenic potential of the genus.

## Materials and methods

### Cell culture and reagents

Immortalized human foreskin keratinocytes (N/TERT HFK) provided by Michael Underbrink (University of Texas Medical Branch) and primary HFK were grown in EpiLife medium (MEPICF500, Gibco), supplemented with 60 μM calcium chloride (MEPICF500, Gibco), human keratinocyte growth supplement (MEPICF500, Gibco), and 1% penicillin-streptomycin (PSL02-6X100ML, Caisson). U2OS were maintained in DMEM supplemented with 10% FBS and 1% penicillin-streptomycin. Zeocin (J67140-XF, Alfa Aesar) was used to induce DSBs (10 μg/mL, 10 min). NU7441 (S2638, Selleckchem) was used to inhibit DNA-PKcs phosphorylation (1 μM) and verify the pDNA-PKcs antibody. SiRNA DNA-PKcs was used to further validate pDNA-PKcs antibody. KU55933 (Sigma-Aldrich, SML1109) was used to validate RAD51 antibody as previously described [71]. CCS1477 (CT-CCS1477, Chemietek) was used to inhibit p300 activity (1 μM). sgRNA/CAS9 plasmids (#136938, Addgene) were used to generate a DSB for next-generation sequencing.

### Immunofluorescence microscopy

Cells were seeded onto either 96-well glass-bottom plates and grown overnight. Cells treated with zeocin for specified time and concentration were fixed with 4% formaldehyde. Then, 0.1% Triton-X was used to permeabilize the cells, followed by blocking with 3% bovine serum albumin. Cells were then incubated with the following antibodies: phospho DNA-PKcs S2056 (ab18192, Abcam, 1:200), RAD51 (ab1837, Abcam, 1:200), RPA70 (ab176467, Abcam, 1:200), cyclin E (4132S, Cell Signalling), or HA-tag (#3724, Cell Signalling, 1:100). The cells were washed and stained with the appropriate secondary antibodies: Alexa Fluor 594 (red) goat anti-rabbit (A11012, Thermo Scientific), Alexa Fluor 488 (green) goat anti-mouse (A11001, Thermo Scientific). After washing, the cells were stained with 10 μM DAPI in PBS and visualized with the Zeiss LSM 770 microscope. Images were analyzed using the ImageJ techniques previously described [72]. Cyclin E intensity was measured for each cell. Average cyclin E intensity of cells grown in media without growth factor for 4 hours was used to define the threshold of cyclin E positive. Colocalized foci appear yellow when green and red channels are merged in ImageJ.

### Flow cytometry

Cells were collected from 10 cm plates, at about 80–90% confluence, by using trypsinization. Cells were washed with cold PBS and fixed with 4% formaldehyde in PBS for 10 min. Then, cells were permeabilized with 0.5% Triton-X for 10 min at room temperature. Cells were stained with anti-RAD51 antibody (ab1837, Abcam, 1:100) and Alexa Fluor 488 goat anti-mouse (A11001, Thermo Scientific,). After washing, cells were resuspended in 200 μL PBS and 30 μM DAPI (4′,6-diamidino-2-phenylindole), and incubated in the dark at room temperature for 15 min. Samples were analysed by a LSRFortessa X20 Flow Cytometer. Flowing software (v2.5.1) was used for data analysis.

### Immunoblotting

After being washed with ice-cold PBS, cells were lysed with RIPA Lysis Buffer (VWRVN653-100ML, VWR Life Science), supplemented with Phosphatase Inhibitor Cocktail 2 (P5726-1ML, Sigma) and Protease Inhibitor Cocktail (B14001, Bimake). The Pierce BCA Protein Assay Kit (89167–794, Thermo Scientific) was used to determine protein concentration. Equal protein lysates were run on Novex 3–8% Tris-acetate 15 Well Mini Gels (EA03785BOX, Invitrogen) and transferred to Immobilon-P membranes (IPVH00010, Fisher Scientific). Membranes were then probed with the following primary antibodies: GAPDH (sc-47724, Santa Cruz Biotechnologies, 1:1000) and phospho DNA-PKcs S2056 (ab18192, Abcam). P300 (sc-48343, Santa Cruz Biotechnologies), pATM (13050S, Cell signaling), ATM (92356S, Cell Signaling), pATR (58014S, Cell signaling), and ATR (2790S, Cell signaling). After exposure to the matching HRP-conjugated secondary antibody, cells were visualized using SuperSignal West Femto Maximum Sensitivity Substrate (34095, Thermo Scientific).

### SgRNA/CAS9 transfection

The sequencing was performed 15 subcultures (passaged 1 to 3) after 8E6 expression in N/TERT immortalized HFKs. HFK cells were plated in 2 mL of complete growth medium in a 6-well plate. Cells were used at 80–90% confluency. 2 μL of plasmid (#JS825, Addgene) was diluted in 200 μL Xfect transfection reagent (631317, Takara). The mixture was incubated at room temperature for 15 min. The transfection mixture was added to each well drop-wise and incubated for 48 hours at 37°C. Cells were harvested for DNA extraction and sequencing.

### Next-generation sequencing

Specific primers were designed to cover 0.1 Mb on each side of the Cas9 target site (6689603–6889603 on Chromosome 12) resulting in a total of 42 primer sets each producing a ~5 Kb overlapping amplicon (S1 Table). Primer sets were pooled based on primer dimerization and annealing temperature compatibility. Genomic DNA was extracted using the MagAttract High Molecular Weight DNA kit (Qiagen) according to the manufacturers’ instructions. The target regions were amplified for each sample using the primer pools coupled with KAPA HiFi Hotstart readymix (KAPA Biosystems) using 20 μM primers as well as a 50/53 ^0^C (Annealing temperature 1/2; S1 Table) and a 5-minute extension time. Primers were removed from PCR amplicons using the Highprep PCR cleanup system (Magbio) as specified by the manufacturer. Libraries were prepared from amplicons with Nextera XT DNA library preparation kit (Illumina) and sequenced on a Nextseq 500 system. A minimum of 100x coverage was targeted for each of the tested samples.

### Sequencing analyses

Raw reads were trimmed for quality and mapped to the target region in CLC genomics workbench v21.0. Trimmed reads were normalized manually transfection efficiency. Normalized reads were assessed for indels and structural variants and normalized for paired read variations in CLC Genomics Workbench v 20.0.4 (Qiagen) using a variant threshold of 5 reads and 100 read coverage (5%). Next generation sequencing of mock transfected cells were used as a reference for determining mutations. Thus. only mutations that does not exist in mock transfected cells were reported. Breakpoints (sites of genomic instability), site-specific variant ratios, insertions, deletions, replacements, inversions and complex (combination of 2 or more genomic changes) were compared between 8E6 and the vector control samples.

### Statistical analysis

All values are represented as mean ± standard error (SE). Statistical differences between groups were measured by using Student’s t-test. p-values in all experiments were considered significant at less than 0.05.

## Supporting information

S1 Fig 8E6 allows RAD51 and pDNA-PKcs foci formation to occur in the same cell. (TIFF)

S2 Fig Colocalized foci are larger than non-colocalized foci. (TIFF)

S3 Fig 8E6 increases colocalization of RAD51 and pDNA-PKcs in HFK cells following UV treatment. (TIFF)

S4 Fig p300 level is detected by immunoblot. (TIFF)

S5 Fig 8E6 increases RPA70:pDNA-PKcs and RAD51:pDNA-PKcs colocalization in primary HFKs. (TIFF)

S6 Fig CCS1477 decreases phosphorylated ATR (pATR) and phosphorylated ATM (pATM). (TIFF)

S7 Fig 8E6 increases the frequency of cyclin A negative cells with RAD51 staining. (TIFF)

S8 Fig Controls were used to determine RAD51 staining and G1 gating in HFK cells. (TIFF)

S9 Fig 8E6 requires CtIP to increase the frequency of Rad51 foci formation in G1. (TIFF)

S10 Fig Controls for NU7441 inhibitor of DNA-PKcs. (TIFF)

S11 Fig RAD51/pDNA-PKcs colocalization occurs at CAS9-induced DSBs in 8E6 expressing HFKs. (TIFF)

S1 Table List of primers used for next-generation sequencing. (CSV)
