# Supplementary figures and images for: Beta human papillomavirus 8 E6 allows colocalization of non-homologous end joining and homologous recombination repair factors
Source: PLoS Pathog. 2022 Feb 11;18(2):e1010275. doi: 10.1371/journal.ppat.1010275 (PMC8836322; doi:10.1371/journal.ppat.1010275)

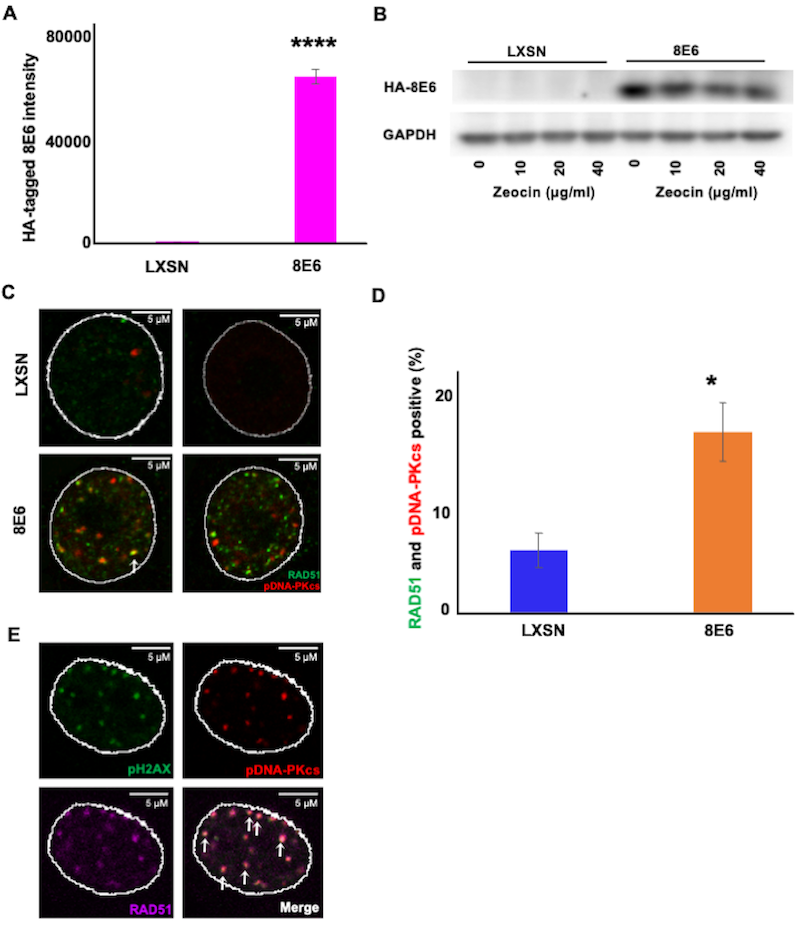

Supplement: S1 Fig — (TIFF) [file ppat.1010275.s001.tiff]

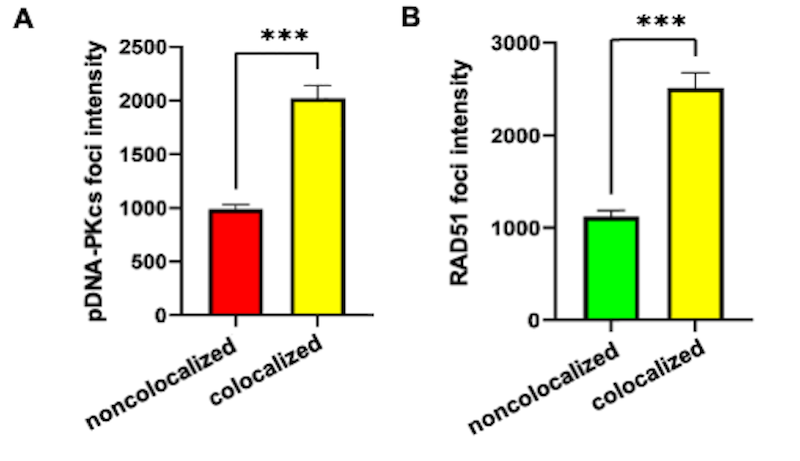

Supplement: S2 Fig — (TIFF) [file ppat.1010275.s002.tiff]

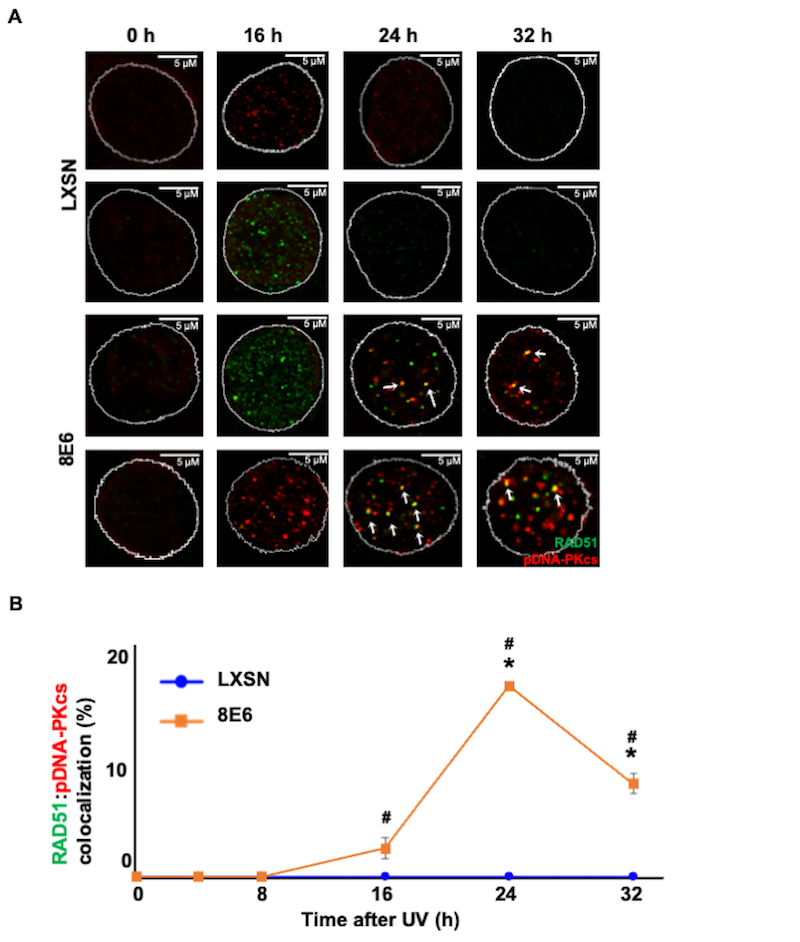

Supplement: S3 Fig — (TIFF) [file ppat.1010275.s003.tiff]

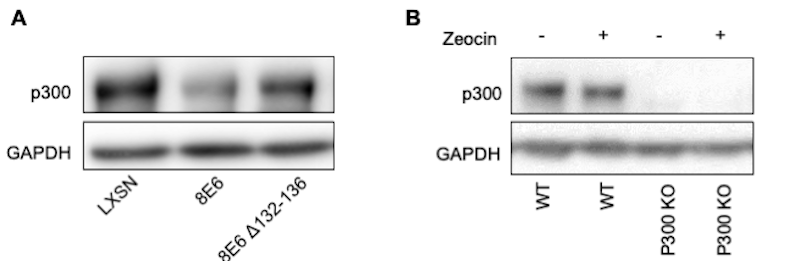

Supplement: S4 Fig — (TIFF) [file ppat.1010275.s004.tiff]

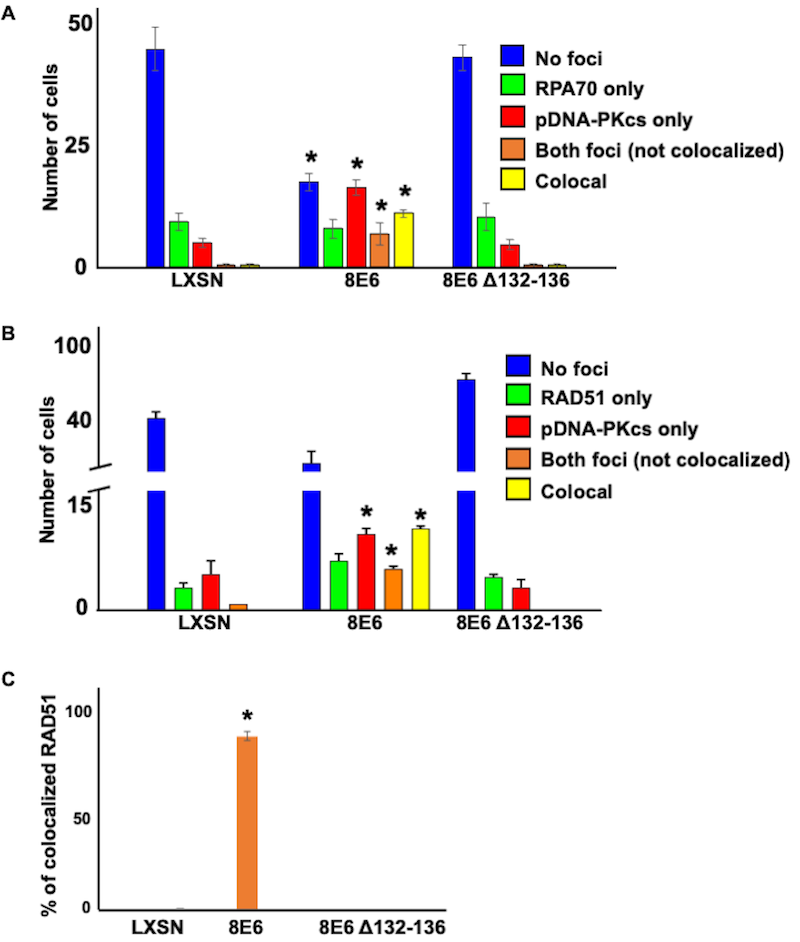

Supplement: S5 Fig — (TIFF) [file ppat.1010275.s005.tiff]

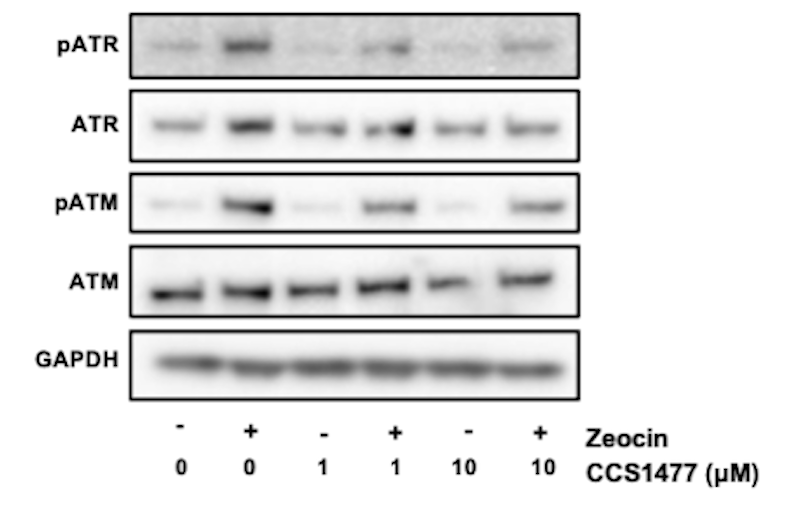

Supplement: S6 Fig — (TIFF) [file ppat.1010275.s006.tiff]

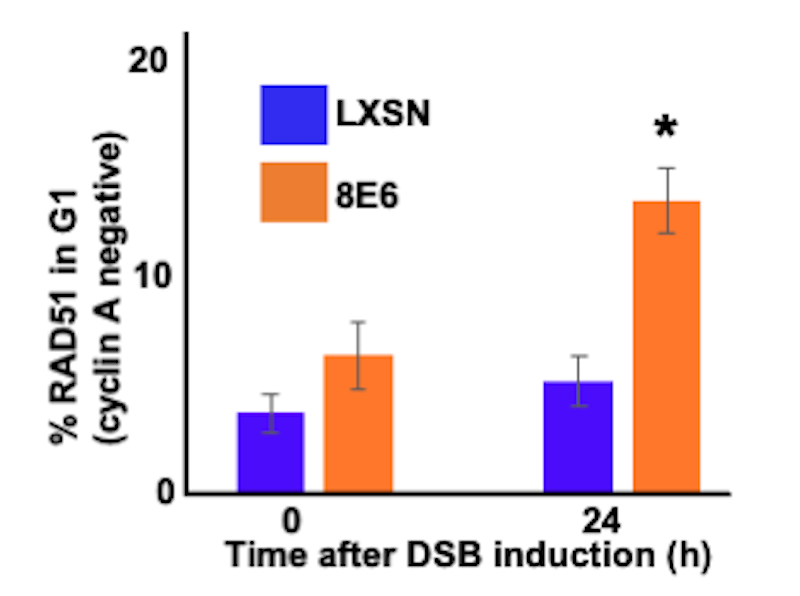

Supplement: S7 Fig — (TIFF) [file ppat.1010275.s007.tiff]

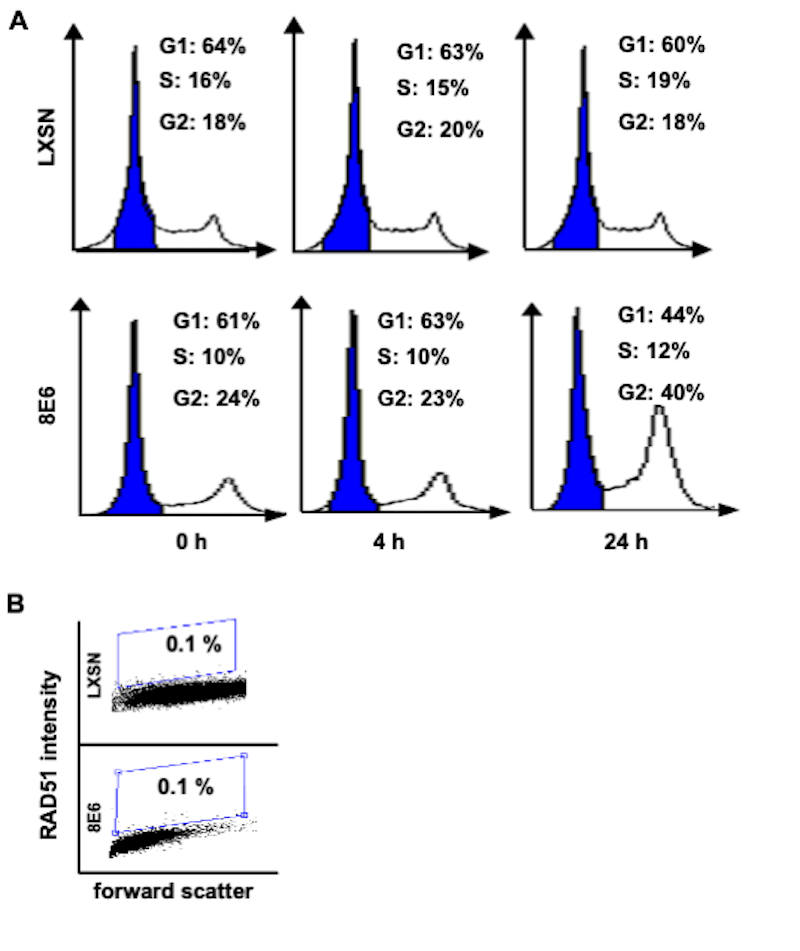

Supplement: S8 Fig — (TIFF) [file ppat.1010275.s008.tiff]

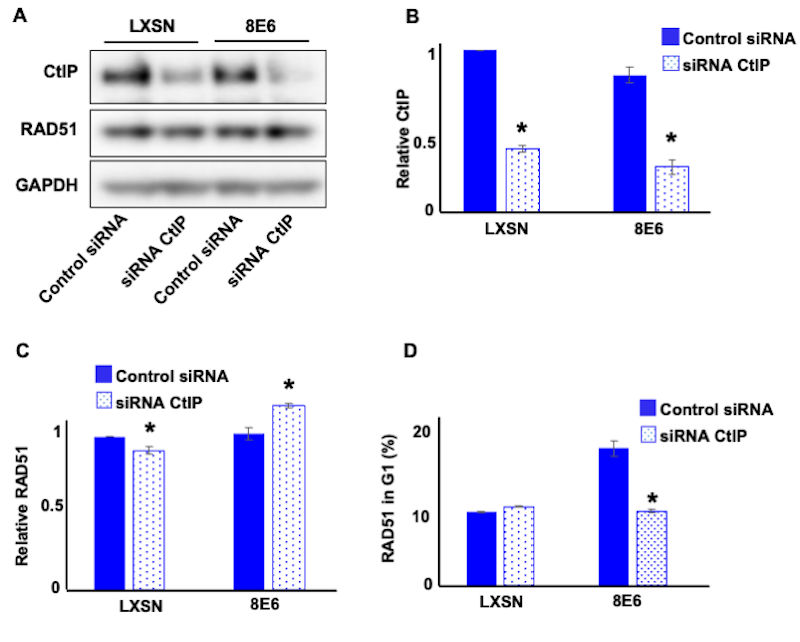

Supplement: S9 Fig — (TIFF) [file ppat.1010275.s009.tiff]

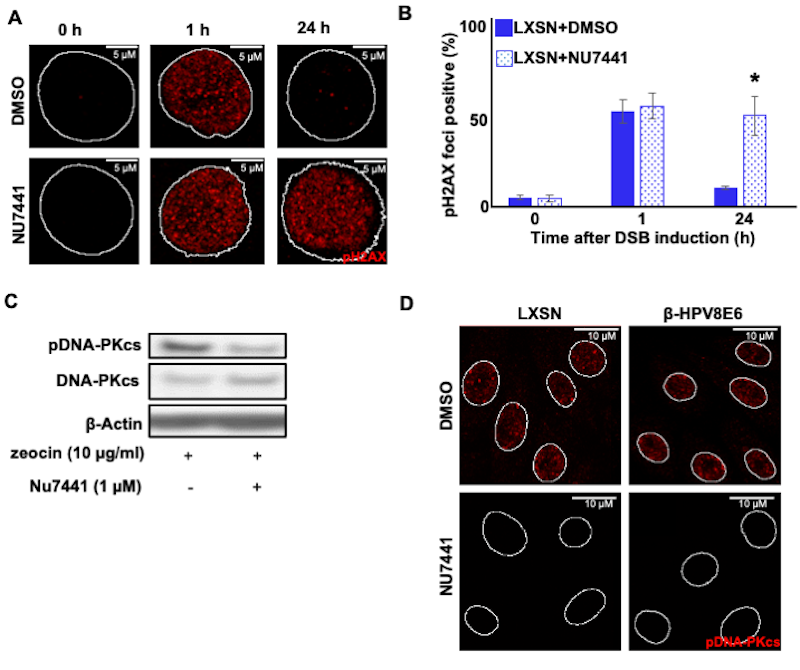

Supplement: S10 Fig — (TIFF) [file ppat.1010275.s010.tiff]

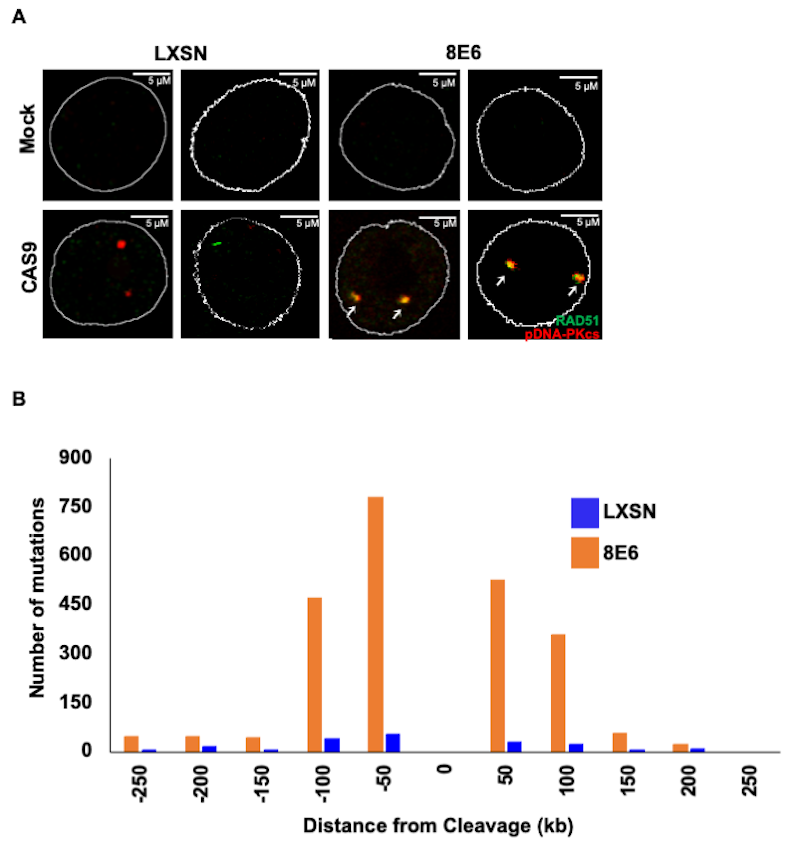

Supplement: S11 Fig — (TIFF) [file ppat.1010275.s011.tiff]
